# Diagnostic Criteria for Gastro-esophageal Reflux Following Sleeve Gastrectomy

**DOI:** 10.1007/s11695-020-05152-5

**Published:** 2021-01-25

**Authors:** Gillian Lim, Yazmin Johari, Geraldine Ooi, Julie Playfair, Cheryl Laurie, Geoffrey Hebbard, Wendy Brown, Paul Burton

**Affiliations:** 1 Department of Surgery, Central Clinical School, Monash University, Level 6, Alfred Centre, 99 Commercial Rd, Melbourne, VIC 3004 Australia; 2 Oesophago-gastric and Bariatric Unit, Department of General Surgery, The Alfred Hospital, Melbourne, Australia; 3 Department of Gastroenterology, Royal Melbourne Hospital and University of Melbourne, Melbourne, Australia

**Keywords:** Sleeve gastrectomy, Gastro-esophageal reflux disease, Diagnostic thresholds, High-resolution esophageal manometry, 24-h ambulatory pH monitoring

## Abstract

**Background:**

Gastro-esophageal reflux disease (GERD) post-sleeve gastrectomy (SG) is a controversial issue and diagnostic dilemma. Strong heterogeneity exists in the assessment of reflux post-SG, and better diagnostic tools are needed to characterize symptomatic reflux. We aimed to determine the discriminant factors of symptomatic reflux and establish diagnostic thresholds for GERD following SG.

**Materials and Methods:**

Patients post-SG were categorized into asymptomatic and symptomatic cohorts and completed validated symptom questionnaires. All patients underwent stationary esophageal manometry and 24-h ambulatory pH monitoring. Univariate and multivariate analyses were conducted to determine the strongest discriminant factors for GERD.

**Results:**

Baseline characteristics of the asymptomatic cohort (*n* = 48) and symptomatic cohort (*n* = 76) were comparable. The median post-operative duration was 7.3 (14.1) vs 7.5 (10.7) months (*p* = 0.825). The symptomatic cohort was more female predominant (90.8 vs 72.9%, *p* = 0.008). Reflux scores were significantly higher in the symptomatic group (36.0 vs 10.5, *p* = 0.003). Stationary manometry parameters were similar, including hiatus hernia prevalence and impaired esophageal motility. The symptomatic cohort had significantly higher total acid exposure, especially while supine (11.3% vs 0.6%, *p* < 0.001). Univariate and multivariate regressions delineated reflux score and supine acid exposure as discriminant factors for symptomatic reflux. The thresholds for distinguishing symptomatic reflux are as follows: reflux score of 11.5 (sensitivity 84.0%, specificity 68.2%) and supine acid exposure of 2.65% (sensitivity 67.1%, specificity 70.8%).

**Conclusion:**

A reflux score of 11.5 or more or supine acid exposure of 2.65% or more should be considered diagnostic in defining symptomatic reflux following SG.

## Introduction

Gastro-esophageal reflux disease (GERD) post-sleeve gastrectomy (SG) is a significant issue. Severe symptoms, need for lifelong medical therapy or revisional surgery [1, 2], and development of Barrett’s esophagus have been reported. Genco et al. and Sebastianelli et al. cited the prevalence of de novo Barrett’s esophagus as 18.8% at 78 months and 17.2% at 145 months post-operative [3, 4]. Given the altered anatomy and incompletely understood physiology of the procedure, it is critical that accurate and specific means of diagnosing pathological GERD are established.

In a 2011 systematic review by Chiu et al., only 4 studies out of 15 reported an increased propensity for reflux post-SG [5]. Systematic reviews in 2016 and 2020 reported an increase in reflux post-operatively, ranging from 19 to 20% with strong heterogeneity among studies on post-SG reflux symptoms [6, 7]. Assessment tools used included subjective symptom reporting and structured questionnaires. Despite the increase in reflux symptoms post-SG in many patients, satisfaction and quality of life were still acceptable and comparable to RYGB [8].

Several mechanisms are theorized to increase GERD following SG including altered lower esophageal sphincter (LES) basal tone [9], hiatus hernia [10], and decreased gastric compliance [11]. Accelerated gastric emptying [12] and sustained weight loss have been posited to produce the opposite effect. In the midst of this debate, however, a consensus framework on the etiology of GERD post-SG has not been established.

Previous studies using esophageal manometry and 24-h pH monitoring have demonstrated variable results but have not yet established diagnostic thresholds. Burgerhart et al. and Gorodner et al. reported increased acid exposure at 193% at 3 months and 102% at 12 months post-operative, respectively [13, 14]. Similarly, Braghetto et al. found persistent elevation of the DeMeester score in symptomatic patients at 5–9 years follow-up [9]. Conversely, Rebecchi et al. found improvement in total acid exposure and DeMeester score 2 years post-SG in patients with preoperative pathological pH profiles, with minimal change in LES basal pressure and esophageal contractility [15].

We hypothesized that the altered anatomy and physiology of SG would require unique, specific criteria to reliably diagnose GERD following SG. We therefore aimed to establish these criteria using esophageal manometry and 24-h pH monitoring.

## Methods

### Patient Selection

Ethics approval was obtained from the Alfred Human Research and Ethics Committee (HREC) no. 380/16 and the Avenue Hospital HREC no. 236. Patients who underwent SG from April 2014 to May 2019 were recruited for this prospective study, and informed written consent was obtained. Laparoscopic SG was performed as previously described [16]. Day 1 post-operative contrast swallow was performed for anatomical definition. All patients underwent a modified diet protocol with gradual transition from liquid to semi-solid diet as well as a proton pump inhibitor for 6 weeks post-operative. Normal diet was instituted after 6 weeks with cessation of the proton pump inhibitor if not clinically required.

- Inclusion criteria: age between 18 and 65 years, at least 6 weeks post-operative with a confirmed anatomically unremarkable sleeved stomach on contrast swallow. This was defined as a tubular-shaped sleeved stomach with no retained stomach and good flow of contrast without anatomical obstruction or leak.
- Exclusion criteria: previous non-bariatric esophago-gastric surgery, pre-existing esophago-gastric motility disorder, and known pre-operative or intra-operative medium to large hiatus hernia.

Participants were categorized based on Visick scoring [17] into asymptomatic (Visick scores I and II) and symptomatic (Visick scores III and IV).

### Data Collection and Outcome Measures

Baseline and post-operative anthropometric data were collected at routine follow-up. Total body weight loss percentage (TBWL%) was defined as percentage of weight loss compared to pre-operative weight. Excess weight loss percentage (EWL%) was calculated as weight loss from pre-operative excess weight compared to ideal weight at BMI 25.

Participants also completed validated symptom questionnaires for reflux [18] and dysphagia [19].

#### Stationary Manometry

A 16-channel silicone nasogastric manometry catheter with a water-perfused system (Mui Scientific, Ontario, Canada) was used with real-time recording facilitated by TRACE!1.2 (written by G Hebbard using LabVIEW, National Instruments, Austin, TX). Participants ceased proton pump inhibitor or H2 antagonist use 10 days prior and were permitted to have a light breakfast and clear fluids the morning of the investigation. A standardized protocol [20] was utilized, consisting of 60 s of supine basal recording, five deep inspirations, ten wet swallows each with 5 ml of water, and five consecutive swallows of 10 ml of water in rapid succession.

**Fig. 1:**
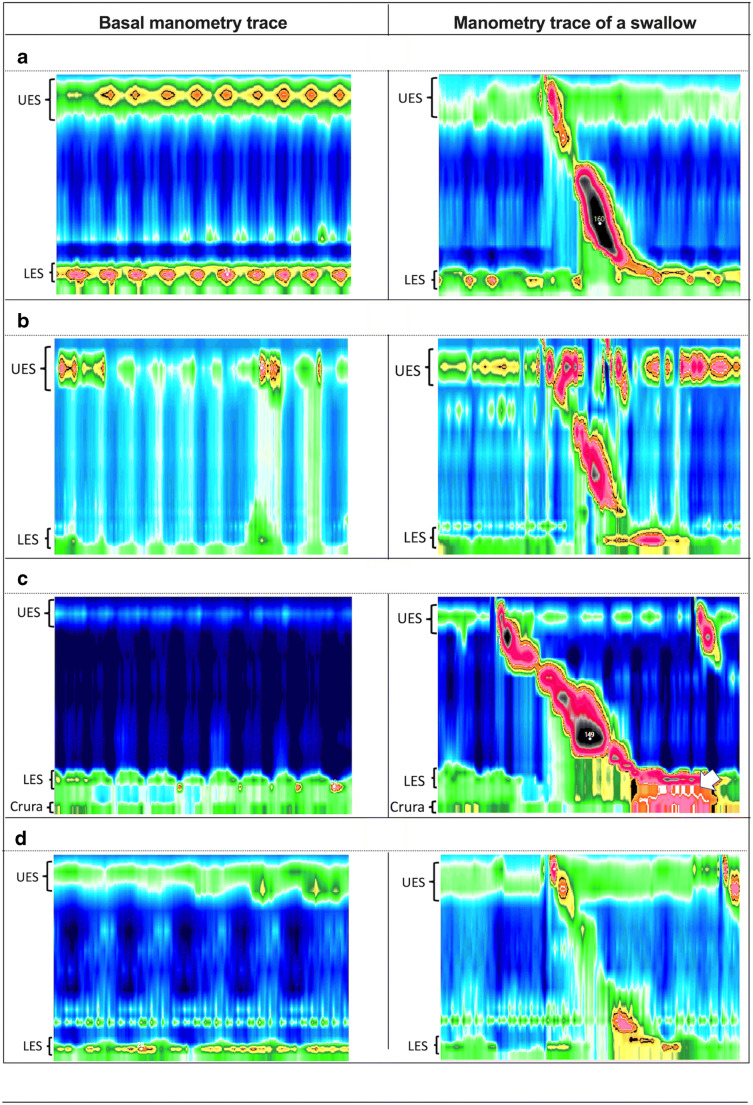
Stationary manometry variables: comparison of basal manometry and manometric trace of a swallow. (**a**) Normal LES basal tone. (**b**) Hypotensive LES basal tone. (**c**) Hiatus hernia (white arrow representing a pocket of high pressure in the hiatus hernia). (**d**) Impaired/uncoordinated esophageal peristalsis

A reference point was drawn from the basal end-expiratory intragastric pressure. Impaired esophageal motility, LES basal pressure, and hiatus hernias were recorded (Fig. 1). Esophageal motility was analyzed according to a published guideline [21]. LES basal pressure was defined as peak end-expiratory LES pressure and LES relaxation percentage as basal pressure at the initiation of swallowing. The crural diaphragm was identified at the axial level of maximal inspiratory pressure. Hiatus hernias were defined as axial separation of the LES and the diaphragm.

#### Ambulatory 24-H pH Monitoring

The pH catheter (Innologic, Australia) was inserted via nasogastric intubation and positioned 5 cm above the LES, which was visualized during manometry. Across 24 h, participants were instructed to consume a normal diet, bar acidic foods. Participants kept a diary of symptoms, food intake, and significant change of posture.

**Fig. 2:**
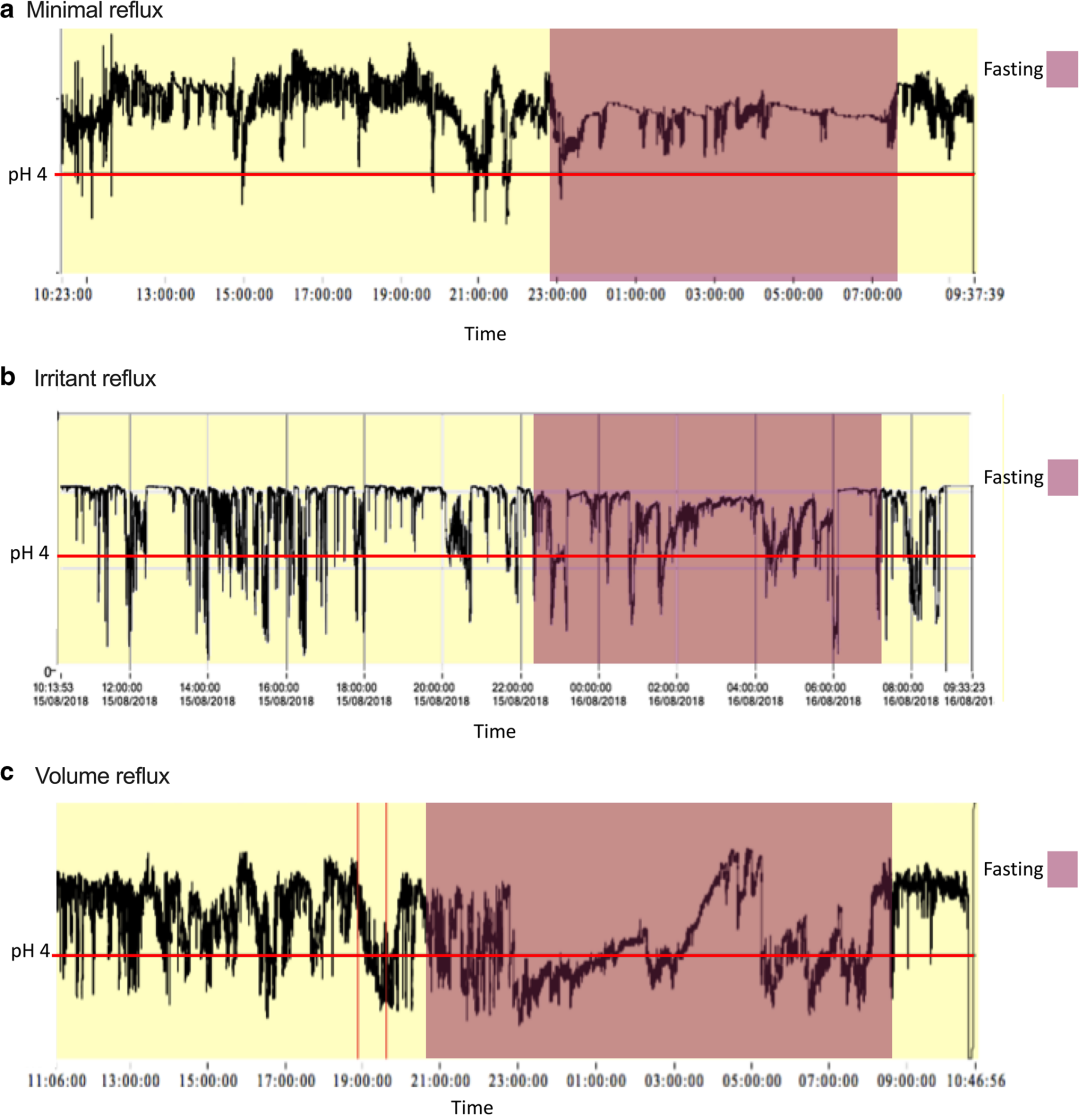
Patterns of reflux on 24-h ambulatory pH monitoring. (**a**) Minimal reflux—minimal acid exposure and reflux events. (**b**) Irritant reflux—multiple reflux events with short duration of acid exposure in both supine and erect states. (**c**) Volume reflux—longer duration of reflux events during the supine state

The 24-h pH data was analyzed using Vanilla pH 1.1 (written by G Hebbard using LabVIEW, National Instruments, Austin, TX). Reflux events were defined as episodes of esophageal pH less than four for 5 or more seconds. Variables collected were the acid exposure, number of events, and duration of acid events. Reflux patterns (Fig. 2) were defined by inspection of 24-h pH traces:

- Minimal reflux—Total acid exposure less than 3% and minimal reflux events
- Irritant reflux—Total acid exposure more than 3% with numerous short reflux events (average duration of each acid events of less than 1 min)
- Volume reflux—Total acid exposure more than 3% with long reflux events (average duration of each acid events of more than 1 min)

### Candidate Variables

Variables considered likely for the diagnosis of GERD post-SG are listed in Table 1. We performed further analysis on clinical, manometric, and pH variables that correlated with the presence of hiatus hernia post-SG.

**Table 1:** Candidate variables for the diagnosis of GERD

Demographic and clinical parameters	24-h pH monitoring parameters	Manometric parameters
Age Gender Preoperative weight and BMI EWL% Revisional SG Duration from surgery Reflux score Dysphagia score	Total esophageal acid exposure Total number of reflux events Mean duration of reflux events Erect esophageal acid exposure Supine esophageal acid exposure Supine reflux event percentage Reflux patterns	LES basal tone LES relaxation percentage Impaired esophageal peristalsis Hiatus hernia

### Data Representation and Statistical Analysis

Univariate binary logistic regression was used to determine the relationship between each variable and the cohorts (asymptomatic valued as 0 and symptomatic as 1). Multivariate binary logistic regression with stepwise backward (Wald) was used to determine the relationship between statistically significant variables in the univariate analysis and cohorts. Omnibus tests of model coefficients were used to determine overall model fit and statistical significance. Nagelkerke *R*^*2*^ method was used to determine how much variation can be explained by the model. The receiver operating characteristic (ROC) curves were created using significant variables from the multivariate regression to determine thresholds that discriminate symptomatic reflux. The area under the curve (AUC) was classified according to Hosmer et al., where AUC more than 0.9 was considered outstanding, between 0.8 and 0.9 excellent, between 0.7 and 0.8 acceptable, and less than 0.7 poor discrimination [22].

Data was compiled using a customized Microsoft Access 2010 database (Microsoft Corporation, Redmond, WA, USA) connected to SQL server. Statistical analysis was performed using SPSS version 26 (SPSS Inc., Chicago, IL, USA) and GraphPad Prism version 8.3.0 (GraphPad Software, San Diego, CA, USA).

## Results

### Cohort Characteristics

A total of 124 participants were included: 48 asymptomatic and 76 symptomatic. The demographic data is displayed in Table 2. Cohorts were mostly demographically similar; however, there was a higher proportion of female participants in the symptomatic cohort. Weight loss was significant and similar between cohorts at median follow-up of 7.4 months. The reflux score was expectedly higher for the symptomatic cohort, but the dysphagia score did not vary significantly between cohorts.

**Table 2:** Characteristics of the asymptomatic and symptomatic cohorts

	Asymptomatic	Symptomatic	*p* value
*N*	48	76	
Age, years	47.6 ± 11.6	44.1 ± 11.4	0.103*
Female gender, *N* (%)	35 (72.9)	69 (90.8)	*0.008*^*#*^
Pre-operative weight, kg	133.4 ± 25.2	126.5 ± 23.8	0.129*
Pre-operative BMI, kg/m^2^	47.5 ± 7.2	45.6 ± 8.0	0.188*
Total body weight loss, %	29.2 ± 17.6	31.6 ± 16.7	0.455*
Excess weight loss, %	53.2 ± 27.4	55.5 ± 25.4	0.650*
Revisional sleeve gastrectomy, *N* (%)	8 (16.7)	16 (21.6)	*0.501*^*#*^
Duration from surgery, median (IQR), months	7.3 (14.1)	7.5 (10.7)	*0.825^*
Adverse gastrointestinal symptoms, median (IQR)			
Reflux 0 = no reflux to 72 = frequent reflux	10.5 (21.5)	36.0 (26.8)	*0.003^*
Dysphagia 0 = no dysphagia to 45 = frequent dysphagia	7.3 (16.5)	12.3 (17.3)	*0.125^*
Stationary manometry			
LES relaxation, median (IQR), %	76.4 (24.2)	68.5 (47.7)	*0.224*
LES basal tone, median (IQR), mmHg	17.1 (15.8)	12.9 (16.0)	*0.112*
Axial separation of LES and diaphragm (hiatus hernia), *N* (%)	16 (33.3)	30 (39.5)	*0.566*
Size of hiatus hernia, median (IQR), cm	3.0 (1.4)	3.5 (1.3)	*0.217*
Impaired esophageal peristalsis, *N* (%)	11 (22.9)	28 (36.8)	*0.085*
24-h pH monitoring			
Total acid exposure, median (IQR), %	4.0 (5.2)	9.2 (13.0)	*< 0.001^*
Number of acid events, median (IQR), *N*	37 (37)	50 (54)	*< 0.001^*
Duration of each acid event, median (IQR), minutes	1.2 (2.6)	2.3 (2.5)	*0.435^*
Erect acid exposure, median (IQR), %	5.0 (7.7)	7.6 (9.1)	*< 0.001^*
Supine acid exposure, median (IQR), %	0.6 (7.0)	11.3 (17.6)	*< 0.001^*
Supine reflux event percentage, median (IQR), %	10.0 (18.6)	22.8 (21.4)	*0.005^*
Reflux patterns Minimal reflux, *N* (%) Irritant reflux, *N* (%) Volume reflux, *N* (%)			*0.001*^*β*^
	*23 (47.9)*	*14 (18.4)*	*< 0.05*
	*11 (22.9)*	*18 (23.7)*	*> 0.05*
	*14 (29.2)*	*44 (57.9)*	*< 0.05*

*Student’s *t* test

^#^Chi-square

^Mann-Whitney

^β^Fisher’s exact test with column proportion comparisons (Bonferroni method)

Stationary manometry revealed similar LES relaxation percentage, LES basal tone, and impaired esophageal peristalsis incidence. The prevalence of hiatus hernias (Fig. 1c) was comparable. There was no hiatus hernia larger than 5 cm in either cohort.

Ambulatory 24-h pH monitoring displayed a markedly high acid exposure profile among symptomatic participants. Erect acid exposure was elevated in the symptomatic cohort, as was supine acid exposure. Symptomatic patients were more likely to demonstrate a volume reflux pattern.

### Univariate and Multivariate Analyses of Distinguishing Factors of Symptomatic Reflux

Table 3 shows the univariate and multivariate binary regression analyses to identify factors delineating symptomatic reflux. From the univariate analysis, gender (OR 3.661), reflux score (OR 1.031), and several 24-h pH variables (OR ranged from 1.017 to 1.221) were significant. Stationary manometry variables did not reach statistical significance in the univariate analysis and were excluded from the multivariate analysis.

**Table 3:** Univariate and multivariate binary logistic regressions of factors associated with symptomatic reflux

Variable	Univariate regression			Stepwise multiple regression*		
	Odds ratio	95% confidence interval	*p* value	Odds ratio	95% confidence interval	*p* value
Age	0.974	0.943–1.005	0.105			
Gender	*3.661*	*1.340–10.001*	*0.011*			
Pre-operative weight	0.988	0.974–1.003	0.130			
Pre-operative BMI	0.969	0.923–1.016	0.189			
Excess weight loss	1.003	0.989–1.017	0.647			
Revisional sleeve gastrectomy	1.379	0.539–3.529	0.502			
Reflux score	*1.031*	*1.001–1.061*	*0.040*	*1.033*	*1.002–1.065*	*0.038*
Dysphagia score	1.050	0.987–1.116	0.123			
Duration of follow-up	0.995	0.976–1.014	0.590			
LES relaxation %	0.989	0.975–1.002	0.107			
LES basal tone	0.987	0.961–1.013	0.310			
Hiatus hernia	1.250	0.583–2.680	0.566			
Impaired esophageal peristalsis	2.061	0.897–4.731	0.088			
Total acid exposure	*1.221*	*1.107–1.347*	*< 0.001*			
Duration of each acid events	1.132	0.889–1.442	0.314			
Number of acid events	*1.017*	*1.007–1.026*	*0.001*			
Erect acid exposure	*1.165*	*1.062–1.279*	*0.001*			
Supine acid exposure	*1.168*	*1.071–1.273*	*< 0.001*	*1.224*	*1.049–1.428*	*0.010*
Supine reflux event percentage	*1.023*	*1.003–1.043*	*0.021*	*1.072*	*1.002–1.065*	*0.038*
Minimal reflux pattern	*0.245*	*0.109–0.552*	*0.001*			
Irritant reflux pattern	1.044	0.444–2.457	0.922			
Volume reflux pattern	*3.339*	*1.544–7.221*	*0.002*			

*Stepwise Backward (Wald) multiple regression performed with gender, total acid exposure, number of acid events, erect acid exposure, supine acid exposure, supine reflux event percentage, reflux score, minimal and volume reflux pattern

Italics refers to statistically significant variables

**Fig. 3:**
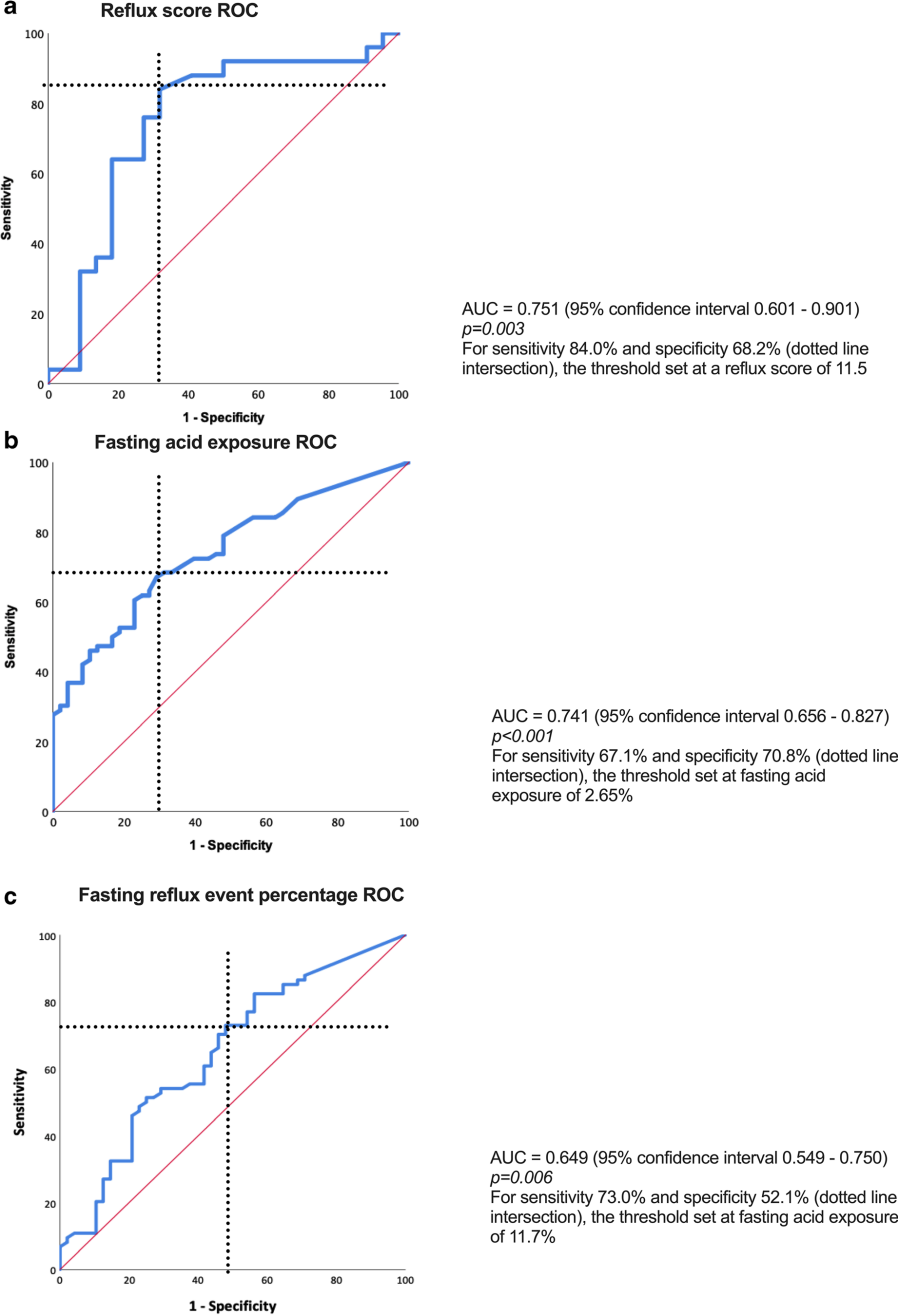
The receiver operating characteristic curves. (**a**) The reflux score, (**b**) supine acid exposure, and (**c**) supine reflux event percentage

The binary regression model was statistically significant (chi-square 28.553, *p* < 0.001). The model explained 60.8% (Nagelkerke *R*^*2*^) of the variance in reflux symptom severity following SG. Only three variables correlated positively and significantly with symptomatic reflux. The *reflux score* produced OR 1.033 (*p* = 0.038), and the discriminant ability of the ROC curve (Fig. 3a) score was acceptable (AUC = 0.751, *p* = 0.003). A threshold of 11.5 distinguished symptomatic reflux with 84.0% sensitivity and 68.2% specificity.

**Fig. 4:**
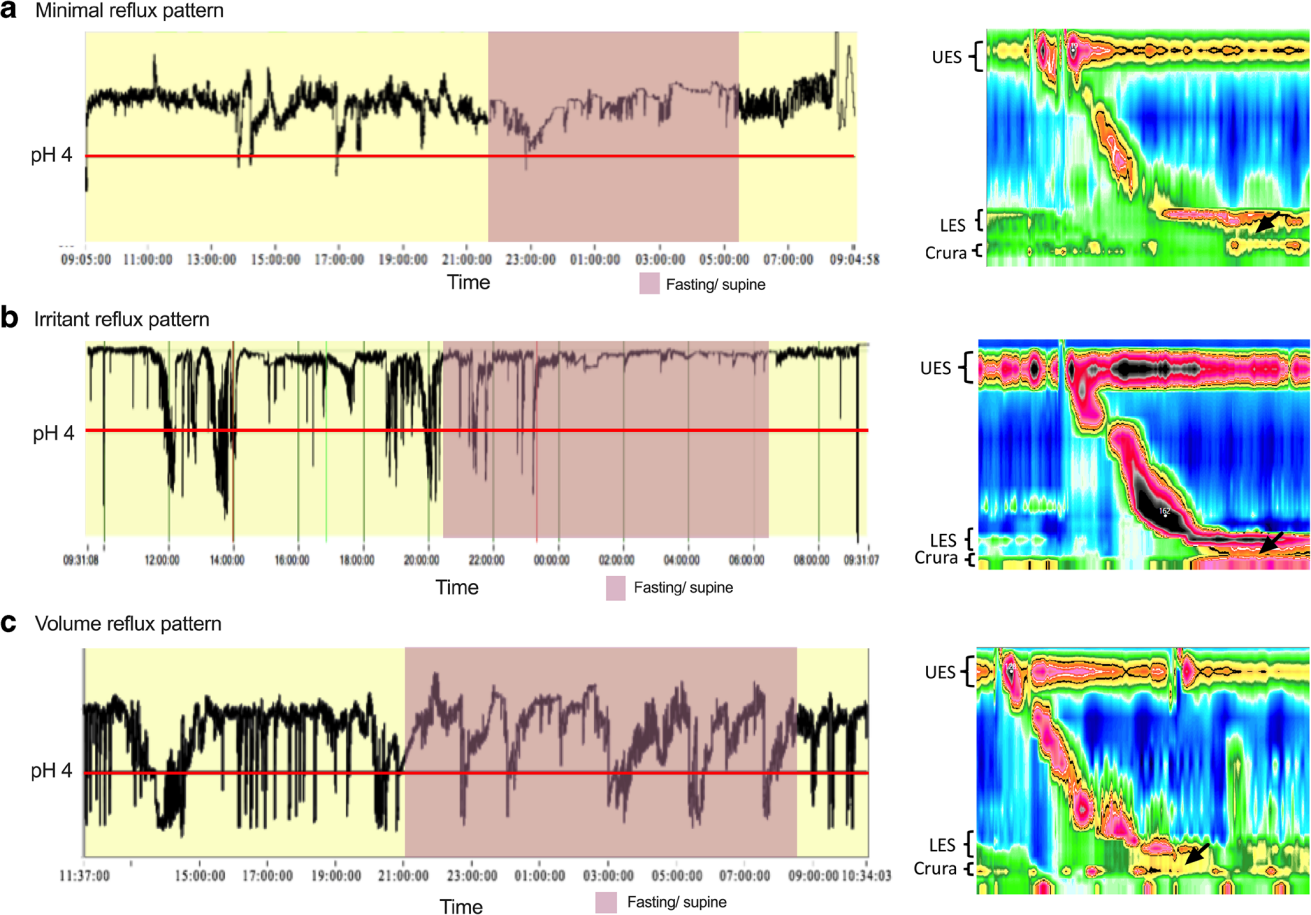
Patterns of reflux in patients with hiatus hernia (24-h ambulatory pH and manometry). (**a**) Minimal reflux. (**b**) Irritant reflux. (**c**) Volume reflux (black arrows representing pockets of high pressure in the hiatus hernia)

From 24-h pH monitoring, higher *supine acid exposure* was associated with an increased likelihood of symptomatic reflux (OR 1.224, *p* = 0.010). The discriminant ability of the ROC curve (Fig. 3b) was also acceptable (AUC = 0.741, *p* < 0.001). A threshold of 2.65% discriminated symptomatic reflux with 67.1% sensitivity and 70.8% specificity. *Supine reflux acid exposure event percentage* correlated positively with symptomatic reflux, with OR 1.072 (*p* = 0.038). However, this variable was a poor discriminator of symptomatic reflux (AUC = 0.649, *p* = 0.006) (Fig. 3c). A threshold of 11.7% distinguished symptomatic reflux with 73.0% sensitivity and 51.2% specificity (Fig. 4).

### Subgroup Analysis: Factors Associated with the Presence of Hiatus Hernia

Univariate analysis (Table 4) showed that several pH variables have significant positive correlation with hiatus hernias: supine acid exposure (OR 1.043, *p* = 0.018) and volume reflux pattern (OR 2.132, *p* = 0.047). Hiatus hernias were negatively correlated with minimal reflux pattern (OR 0.301, *p* = 0.012). The association between hiatus hernias and symptomatic reflux was not statistically significant (OR 1.250, *p* = 0.566).

**Table 4:** Univariate binary logistic regressions of factors associated with the presence of hiatus hernia post-sleeve gastrectomy

Variable	Univariate regression		
	Odds ratio	95% confidence interval	*p* value
Symptomatic reflux	1.250	0.583–2.680	0.566
Age	1.031	0.999–1.065	0.061
Gender	1.882	0.629–5.626	0.258
Pre-operative weight	0.997	0.982–1.012	0.703
Pre-operative BMI	1.010	0.962–1.060	0.683
Excess weight loss	0.997	0.983–1.012	0.710
Revisional sleeve gastrectomy	0.983	0.390–2.479	0.983
Reflux score	1.011	0.987–1.035	0.370
Dysphagia score	1.033	0.973–1.033	0.286
Duration of follow-up	0.995	0.976–1.014	0.590
LES relaxation %	*0.984*	*0.971–0.998*	*0.022*
LES basal tone	0.980	0.951–1.011	0.199
Hiatus hernia	1.250	0.583–2.680	0.566
Impaired esophageal peristalsis	2.061	0.897–4.731	0.088
Total acid exposure	*1.064*	*1.005–1.126*	*0.034*
Duration of each acid events	0.998	0.799–1.247	0.986
Number of acid events	*1.006*	*1.000–1.012*	*0.046*
Erect acid exposure	1.029	0.964–1.098	0.392
Supine acid exposure	*1.043*	*1.007–1.081*	*0.018*
Supine reflux event percentage	1.014	0.997–1.031	0.0.96
Minimal reflux pattern	*0.301*	*0.119–0.764*	*0.012*
Irritant reflux pattern	1.204	0.514–2.822	0.669
Volume reflux pattern	*2.132*	*1.010–4.499*	*0.047*

Italics refers to statistically significant variables

A stepwise backward multivariate model revealed significant positive correlation between supine acid exposure and hiatus hernia (OR 1.049, 95% CI 1.010–1.090, *p* = 0.013). The model lacked generality (Nagelkerke *R*^*2*^ 0.082) despite being a statistically significant model (chi-square 7.227, *p* = 0.007).

#### A Summary of Our Findings Is Listed in Box 1

Box 1 Practical application for the diagnosis of GERD following sleeve gastrectomy

**Table:** 

Objective diagnostic criteria of symptomatic reflux: 24-h ambulatory pH: Supine acid exposure more than 2.65% Reflux score (18) more than 11.5 out of 72 Important considerations: Clinical - Reflux scores are generally low-post-SG: the median reflux score of asymptomatic patients was 10.5 (IQR 21.5) out of 72 24-h ambulatory pH monitoring - Higher acid exposure is expected post-SG: the median total acid exposure of asymptomatic patients was 4.0% (IQR 5.2) Manometry - Hiatus hernias, LES incompetence, and impaired peristalsis may play a role in the severity of reflux following SG - No strong correlation between hiatus hernias and symptomatic reflux was found on subgroup analysis. However, hiatus hernias were associated with higher supine acid exposure	

## Discussion

We have identified two significant factors that can be reliably used to diagnose symptomatic reflux post-SG. These differ markedly from established criteria used to diagnose GERD in patients with an anatomically normal stomach. We demonstrated that a moderate increase in esophageal acid exposure is expected following SG. Despite the presence of elevated esophageal acid exposure, many patients did not experience significant reflux symptoms. Using defined normal values, we were able to identify a threshold supine acid exposure value for abnormal esophageal acid levels. Additionally, we identified a cut-off value that defined abnormal GERD post-SG using an established reflux score [18].

Our findings are in line with aspects of previous literature that have focused on discriminating symptomatic reflux. Reflux symptoms and abnormal 24-h pH score have been previously identified as significant factors in the identification of symptomatic reflux following laparoscopic fundoplication and were subsequently found to be strong predictors of success [23].

Significant esophageal acid exposure can be present post-SG in the absence of significant symptoms. The mean esophageal acid exposure in asymptomatic patients was high (median 4.2%) compared to normal laboratory values [24]. Despite the significant elevation in acid exposure in symptomatic patients, it overlapped substantially with acid exposure for asymptomatic patients.

Supine acid exposure is a significant discriminant factor, and exposure of more than 2.65% should be considered diagnostic of GERD post-SG. This threshold was found to have 84% sensitivity and 68% specificity. Meeting this criterion should be considered a marker of substantially disrupted physiology given that minimal, if any, nocturnal esophageal acid exposure would be expected normally.

The patient-reported reflux questionnaire developed by Anvari et al. was initially developed and validated for patients undergoing Nissen fundoplication. The scoring system has been used to describe the SG population since then [14, 25]; however, a definition of abnormal GERD in this population had not been established. An objective reflux score threshold of 11.5 defined symptomatic reflux, with reasonable sensitivity of 84% and specificity of 68.4%.

Notably, manometric variables, including LES basal tone and relaxation, did not discriminate between the two cohorts. This result appears to contradict previous studies by Braghetto et al. who found decreased LES resting pressure in symptomatic post-SG patients at 5 years [26] and 8–10 years [9].

Hiatus hernias (no more than 5 cm) post-SG were also not found to be a significant discriminant factor of symptomatic reflux but were associated with higher acid exposure. Previous studies have implicated hiatus hernias as a significant factor in symptomatic GERD following SG, and consequently that reflux improves with hiatus hernia repair [10, 27]. While these findings require objective validation, our data adds considerably to current literature. We would suggest that the presence of objectively quantified GERD based on physiological testing and coexistence of a hiatus hernia would more strongly predict a therapeutic response. Conversely, a patient presenting with symptoms in the absence of objective GERD may not benefit from intervention.

The limitations of this study include the single-center nature of the study, which limits the sample size to an extent. On the other hand, we felt this aspect provided streamlined decision-making and management in terms of patient selection for SG and pre-operative and post-operative care. Secondly, the absence of data on pre-operative gastroscopy and GERD precludes the evaluation of correlation between pre-operative and post-operative GERD, which is outside the scope of this study. We also did not evaluate potential post-operative confounding factors of reflux such as alcohol use and cigarette smoking.

Our future endeavors will focus on validating the findings in a separate SG cohort, including multicenter evaluation, which is required before widespread acceptance and generalization can occur. We will also investigate other aspects of symptomatic reflux post-SG including the influence of esophageal sensation, visceral hypersensitivity, and duodeno-gastric reflux. These criteria should also be assessed to determine their role in predicting response to treatment of GERD post-SG or need for further intervention and re-operation. Ultimately, this pursuit will be of considerable value to better understand the mechanisms of esophageal transit and pathophysiology of reflux following SG.

We have proposed threshold values for diagnosing abnormal reflux using both an objective questionnaire and 24-h pH monitoring. Moderately elevated acid exposure appears inherent to the procedure; however, significant elevation of supine acid exposure was the key pathological feature. We propose the use of an objective questionnaire and ambulatory pH when confronted with post-SG patients with reflux symptoms, especially when deciding whether this warrants further intervention and surgical management for GERD. Hopefully, this will assist substantially in the management and decision-making in reflux following SG.
